# Hippocampus Segmentation Based on Local Linear Mapping

**DOI:** 10.1038/srep45501

**Published:** 2017-04-03

**Authors:** Shumao Pang, Jun Jiang, Zhentai Lu, Xueli Li, Wei Yang, Meiyan Huang, Yu Zhang, Yanqiu Feng, Wenhua Huang, Qianjin Feng

**Affiliations:** 1 Guangdong Provincial Key Laboratory of Medical Image Processing, School of Biomedical Engineering, Southern Medical University, Guangzhou, 510515, China; 2 School of Basic Medical Science, Southern Medical University, Guangzhou, 510515, China

## Abstract

We propose local linear mapping (LLM), a novel fusion framework for distance field (DF) to perform automatic hippocampus segmentation. A k-means cluster method is propose for constructing magnetic resonance (MR) and DF dictionaries. In LLM, we assume that the MR and DF samples are located on two nonlinear manifolds and the mapping from the MR manifold to the DF manifold is differentiable and locally linear. We combine the MR dictionary using local linear representation to present the test sample, and combine the DF dictionary using the corresponding coefficients derived from local linear representation procedure to predict the DF of the test sample. We then merge the overlapped predicted DF patch to obtain the DF value of each point in the test image via a confidence-based weighted average method. This approach enabled us to estimate the label of the test image according to the predicted DF. The proposed method was evaluated on brain images of 35 subjects obtained from SATA dataset. Results indicate the effectiveness of the proposed method, which yields mean Dice similarity coefficients of 0.8697, 0.8770 and 0.8734 for the left, right and bi-lateral hippocampus, respectively.

The accurate and reliable segmentation of deep brain structures, such as the hippocampus, in magnetic resonance (MR) images has gained considerable scientific attention because of the widespread use of MRI. The segmentation of deep brain structures is a key requirement for the assessment, treatment, and follow-up of various mental disorders^1^,^2^. The hippocampus is located in the medial temporal lobe, which is the site of structural and functional pathologies in mental illnesses. Changes in the size and shape of the hippocampus are closely related to Alzheimer’s and other diseases. Morphological analysis and shape comparisons of the hippocampus from healthy and diseased subjects can identify abnormal deformations; such findings can facilitate possible biomarker identification, prognosis and diagnosis of diseases, and optimum treatment identification^3^,^4^,^5^. Hippocampus segmentation should be conducted for these applications.

The manual segmentation of the hippocampus from MR images is tedious, time-consuming, susceptible to human errors, nonreproducible, and expensive. Automatic segmentation offers reasonable promises, but remains challenging because of noise, limited resolution, and partial volume effect, resulting in weak boundaries of the hippocampus in MR images^4^,^6^. At present, the segmentation accuracy of the hippocampus remains relatively low^7^,^8^,^9^,^10^.

Atlas-based segmentation is a powerful and popular technique for automatic delineation of structures in volumetric images^9^,^11^,^12^,^13^,^14^,^15^,^16^,^17^,^18^, especially the hippocampus^18^,^19^,^20^. Atlas-based methods are initiated by registering an atlas with the target image to be segmented. The manual label of the atlas associated with the training images is thus propagated to the target image by using the mapping determined during the registration. The quality of atlas-based segmentation is affected by the bias and the accuracy of registration, as well as the label fusion method.

Multiple atlases can be separately registered to the target image to avoid biased registration^7^,^10^,^19^,^20^,^21^,^22^,^23^,^24^,^25^. The corresponding label of each atlas is warped to the target image space through the deformation field derived from the registration procedure. Combining the warped labels from all atlases generates a fused label as the segmentation of the target image. Several studies demonstrated that multi-atlas segmentation methods significantly outperform schemes that use a single atlas^7^,^10^,^19^,^20^,^21^,^22^,^23^,^24^.

Patch-based label fusion methods have been proposed to alleviate the dependence of the accurate registration^21^,^26^. In these methods, the atlases only need to be aligned to the target image space through linear registration. The labels of each patch of the target image are calculated by fusing the labels of similar patches located in the surrounding region in the aligned atlases. The patch-based method has demonstrated promising segmentation results without need for accurate non-rigid registrations.

Another approach to further improve the quality of multi-atlas based segmentation is to develop more accurate and robust label fusion methods. The most straightforward label fusion method is the majority vote on a per-voxel basis^18^. A recent work demonstrated that weighted averaging can be used to improve the quality of segmentation^19^. This approach suggests that an atlas which bears similarities with the target image should carry more weight during label fusion. Most existing label fusion methods are based on weighted voting, in which each atlas contributes to the final solution according to a nonnegative weight; in this method, atlases that bear similarities with the target image receive larger weights^10^. Among weighted voting methods, those that derive weights from local similarity between the atlas and target, and thus allow the weights to vary spatially, have been the most successful in practice. Another popular approach is called simultaneous truth and performance level estimation (STAPLE), which uses an expectation-maximization (EM) approach to obtain the best possible final segmentation^27^. Spatial STAPLE is an extension of the traditional STAPLE framework that enables the estimation of a smooth spatially varying performance level field instead of global performance level parameters and has been shown to provide robust and accurate multi-atlas segmentations^20^. For the patch-based label fusion method, the weights of the fused labels are calculated using a non-local means approach^28^ or local linear representation-based method^26^.

This study is an expansion of the preliminary research^29^, which demonstrated that the use of distance field (DF) improved the accuracy of hippocampus segmentation. In ref. 29, the image patches and DF patches were assumed to be located on different nonlinear manifolds, and the mapping between these manifolds approximated a diffeomorphism under a local constraint. Based on the two assumptions, a distance field fusion (DFF) method was proposed to perform hippocampus segmentation. This method produced promising segmentation results, but two drawbacks were identified in ref. 29. First, training subjects need to be non-rigidly registered to each test subject, which require large memory and complicated computation. Second, high computation costs were required in constructing a dictionary because different test samples use distinct dictionaries.

In this paper, we propose a method based on local linear mapping (LLM) to segment the hippocampus from the MR brain image. The present study achieved the following improvements compared with the preliminary version^29^.

First, we use a fixed dictionary instead of an adaptive dictionary to estimate the DF of the target object (hippocampus in this study). Non-rigid registration is not required for the test image thereby reducing computation and memory costs. The MR image patches and DF patches are assumed to be located on different nonlinear manifolds, and the mapping between these two manifolds is differentiable and linear under a local constraint. The fusion weights of the DF patches can be deduced from the weights of MR image patches. Based on these assumptions, a compact dictionary is constructed via the k-means cluster method. The DFs of test samples are predicted using LLM.

Second, a novel confidence-based weighted average (CWA) method is proposed to merge the overlapped DF patch prediction for each test sample. In CWA, the weight used to predict the DF value of a point is dependent on a residual of local linear representation, wherein large residual indicates less weight and vice versa. The proposed method is evaluated on 35 subjects, which include 20 training subjects, 5 optimization subjects and 10 test subjects. Results show that the proposed method can generate a more promising hippocampus segmentation than that in existing methods^18^,^20^,^27^,^30^,^31^.

The rest of this paper is organized as follows. In Section “Datasets”, we describe the datasets. In Section “Methods”, we show the details of the proposed LLM. In Section “Experimental results”, we present the experiments and results. In Section “Discussion”, discussion is provided.

## Datasets

Our dataset is obtained from SATA Segmentation Challenge Dataset (https://my.vanderbilt.edu/masi/workshops/). We use the subdataset in the dataset to perform the experiments. The subdataset consists of 35 subjects. Each subject includes a T1-weighted MR brain image and a manually delineated label image. The size of the voxels of the images is 1 × 1 × 1 mm^3^. Each image consists of several slices with the resolution of 256 × 256 (pixels) and the numbers of slices range from 261 to 334. We randomly select 20 subjects as training dataset, 5 as optimization dataset and 10 as test dataset.

## Methods

**Figure 1:**
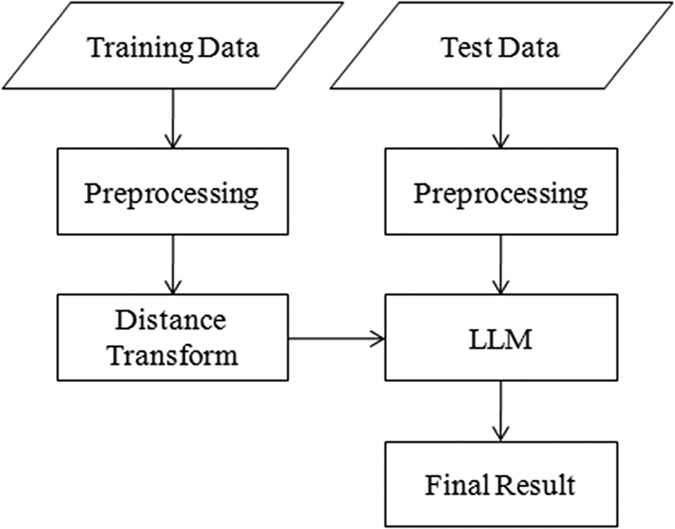
Framework of the proposed method.

The proposed method contains three parts, namely, preprocessing, distance transform, and LLM-based segmentation. The framework of the proposed method is shown in Fig. 1.

### LLM

#### Basic idea of LLM

Given a set of MR images and corresponding DF images associated with the hippocampus, we aim to predict the DF for the test image. The segmentation problem is described as follows. Given a training dataset 
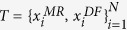
 which consists of *N* MR/DF image patch pairs, we want to calculate the patch 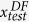 of a test MR image patch 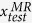. We construct a compact dictionary 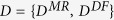 through 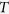 to reduce computation and memory costs caused by the tremendous size of 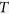. 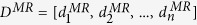 and 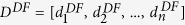 denote the MR dictionary and the DF dictionary, respectively. The dimension of atom vector 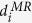 equates to that of 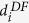. The proposed LLM is based on two assumptions.

***Assumption I:** Image patches from MR image and DF are located on different nonlinear manifolds, and a patch can be approximately represented as a linear combination of several nearest neighbors from its manifolds.*

***Assumption II:** Under a local constraint, the mapping from MR manifold to DF manifold* 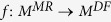 *is differentiable and linear.*

Assumption I was verified in many studies^26^,^32^,^33^. In the present study, manifolds 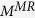 and 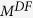, which respectively denote the manifolds of MR and DF, are assumed to be spanned by patches in dictionary 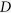. Test image patch 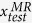 can be linearly represented as follows:


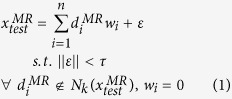


where *ε* is the reconstruction error of sample 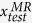 and *τ* is a small non-negative constant. 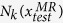 is a set that consists of the *k*-nearest neighbors of sample 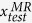 in dictionary 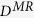.

Based on Assumption II, a local region on manifold *M*^*MR*^ can be mapped to a local region on manifold *M*^*DF*^, and the mapping *f* between these two local regions is linear. The DF of the test patch 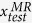 can be calculated as


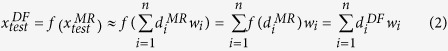


Equation (2) shows that the weight in the original MR image patch space can be transformed to the DF patch space under Assumption I and II. The locality of the sample space is crucial for the rationality of Assumption II. The locality in the sample space means only in the local regions on manifolds *M*^*MR*^ and *M*^*DF*^, *f* is linear and Eq. (2) can be deduced. To maintain the locality of the sample space, we need to gather sufficient samples. Thus for each test sample, dense samples span its local region. The drawback of this approach is the need for a large training dataset. Such a requirement leads to high computation and memory costs. In addition, an appropriate local linear representation method should be selected to solve Eq. (1) in a local region (detailed in Section “Local linear representation”). In this paper, a dictionary construction method based on k-means cluster is proposed to reduce computation and memory costs.

#### Dictionary construction

A dictionary can be constructed using original training dataset *T*. However, numerous training samples possibly produce a large dictionary which contains redundant information and requires large memory and computation costs. Previous studies^26^,^34^ indicated that the k-means cluster method produces a representative dictionary for local linear representation. To reduce computation and memory costs, we use the k-means method to cluster the MR training samples and the cluster centers are selected as the atom vectors of MR dictionary *D*^*MR*^. The atom vector of *D*^*MR*^ is denoted as:


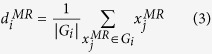


where *G*_*i*_ is a sub-set, and each 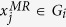 is closest to 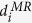 in *D*^*MR*^, i.e. 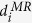 is the cluster center of *G*_*i*_, and 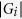 is the size of *G*_*i*_.

In Eq. (3), 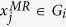 is the neighbor of 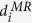. Based on Assumption II, mapping *f* from manifold *M*^*MR*^ to *M*^*DF*^ is locally linear. Therefore, the atom vector of DF dictionary *D*^*DF*^ is denoted as:


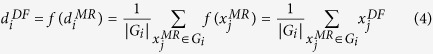


where 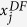 is the DT patch that corresponds to 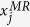.

#### Local linear representation

Several methods have been proposed to represent a test sample linearly by combining training samples. Sparse coding with L_1_ LASSO^35^ emphasizes the sparsity of coefficients. This approach represents a test sample with the least training samples and minimal construction error. Locality-constrained linear coding (LLC)^36^ emphasizes the locality rather than sparsity. This method represents a test sample using several training samples located in a local region around the test sample. Compared with LLC, local anchor embedding (LAE)^37^ adds a non-negative constraint to the coefficients to ensure that the test sample is represented by the convex combination of its closest neighbors and further enhances locality. LLC is selected in local linear representation because it outperforms LAE. According to our context, the cost function of LLC is defined as follows:


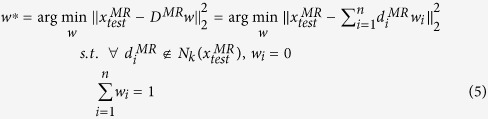


where 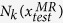 is a set which is composed of the *k*-nearest neighbors of the test sample 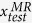 in 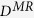, and 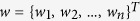 is the coefficient vector of *D*^*MR*^.

#### DF prediction and hippocampus segmentation via CWA

The corresponding patch 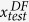 of test patch 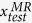 can be predicted using Eq. (2). We slide the patch across the test image step by step, ensuring that patches overlap. For a point *p* in the test image, we can obtain a series of predicted DF values from the overlapped patches. In ref. 29, the average of these predicted values was used as the final estimator for point *p*. The overlapped patches around the point *p* equally contributed to the DF prediction of *p*. However, the confidences of DF prediction from different patches around *p* are different, which result in various contributions for DF prediction. To address this issue, we propose a novel CWA strategy to predict the DF value of *p*. The weighted average of DF values predicted from overlapped patches around *p* is utilized as the final estimator for *p*, and the weights for overlapped patches are calculated by the confidence of DF prediction. We evaluate the confidence by the residual of local linear representation, i.e., larger residual indicates less confidence and vice versa. Based on this analysis, the weight of the point *u* in the patch 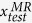 centered at point *p* is calculated as follows:


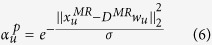


where *w*_*u*_ is the coefficient vector calculated via Eq. (5); 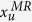 denotes the patch centered at point 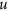; 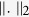 is the L2 norm that indicates the residual of local linear representation; *σ* is a decay parameter. When *σ* is small, only a few more confident patches contribute to DF prediction for *p*. When *σ* is large, all patches around *p* tend to have similar weights and the prediction is similar to a classical average. The value of *σ* should depend on the residuals of the local linear representation of patches centered at points around *p*. When the reconstruction residual of a patch center at a point around *p* is extremely small, *σ* should be decreased to reduce the influence of other patches. By contrast, when the reconstruction residuals of patches centered at points around *p* are extremely large, *σ* should be increased to relax the selection. To achieve the automatic selection of *σ*, we introduce the local adaptation of *σ*^21^ as follows:


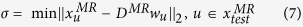


We then obtain the DF value for point *p* using the following formula:


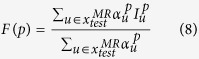


where 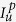 denotes the DF value of *p* estimated by patch 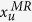 using Eq. (2). The label of point *p* can be calculated as


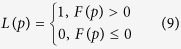


### Preprocessing

The intensity of MR images is normalized to remove variation in image intensity caused by different coordinate systems, head positions, non-uniformity of image intensity, and other artifacts. The BET approach^38^ is applied to remove the skull in MR images; the N4 algorithm^39^ is utilized to remove the bias field artifacts from MR images. All training MR images were non-rigidly registered to a randomly selected MR image via DRAMMS^40^; the average of wrapped MR images and label images are used as average template and average label template, respectively. For each test MR image, the average template was linearly registered to the test MR image via FLIRT^41^ using default parameters. The wrapped average label template is utilized to extract ROI around the hippocampus; as such, computational burden is reduced. Training and test samples are extracted in the original image space.

### Distance Transform

**Figure 2:**
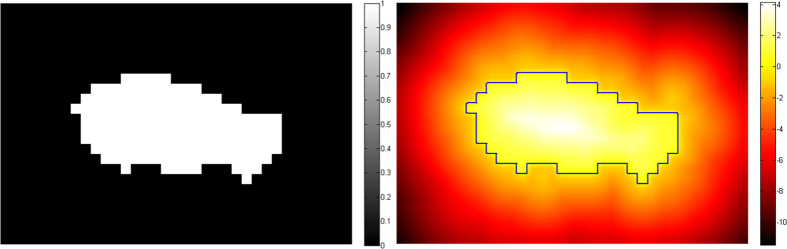
The left column is the label image of the hippocampus; the right column is the DF corresponding to the label image, and the blue contour is the boundary of the hippocampus.

The absolute value of DF at a point *p* denotes the distance between *p* and the closest point from the boundary of the target (i.e. hippocampus). The sign denotes whether the point *p* belongs to the hippocampus. A positive sign indicates that *p* is inside the boundary of the hippocampus and vice versa. Figure 2 shows an example of the DF of a hippocampus image. The DF patch provides the label information and the distance information from the boundary. We used the method proposed by Maurer^42^ to calculate the DF of a label image.

### Summary of the Proposed Method

We provide a pseudo-code in Algorithm 1 to illustrate the proposed method.


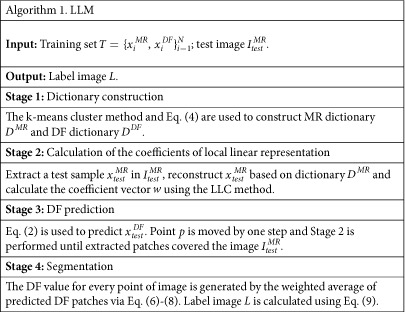


## Experimental Results

We applied the proposed method to 35 subjects. A total of 20 subjects comprised the training dataset, 5 subjects comprised the optimization dataset, and 10 subjects were test dataset. The training dataset was used to construct the dictionary, and the optimization dataset was utilized to optimize parameters, which were used to perform the subsequent experiments using the test dataset to evaluate the performance of the proposed method. The number of nearest neighbors in LLC and LAE was 30, and the step of sliding the patch was 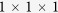 mm^3^ in all experiments.

To evaluate the performance of the methods, we used Dice similarity coefficient (DSC) as the quantitative metric^34^, which is defined as:


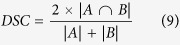


where *A* and *B* denote the voxel sets of the segmentation result and ground truth, respectively. DSC was used to measure the similarity between the ground truth and the automatic segmentation results.

A set of experiments were presented to evaluate the performance of the proposed method. These experiments include (1) parameter choices, (2) contribution of CWA, (3) comparison with state-of-the-art methods. A PC with Intel Xeon E5-2620 2.0 GHz processor and 96GB RAM was used as workstation. Our algorithm was implemented in MATLAB 2012b using single thread. Dictionary construction in the training step lasted for 5 h. This dictionary contained 70,000 atoms and 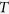 was extracted from a training dataset composed of 20 subjects. In the test step, the processing time was approximately 10 min, including 6 min in preprocessing and 4 min in segmentation.

### Parameter Choices

#### Selection of the number of training subjects

**Figure 3:**
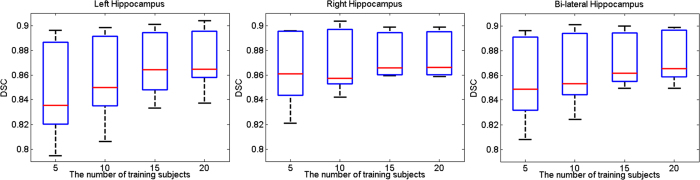
DSC for left, right and bi-lateral hippocampus segmentation over different numbers of training subjects.

Figure 3 illustrates some of the parameter selection experiments, in which the numbers of training subjects varied from 5 to 20 with an interval of 5. In these experiments, the sizes of patch and dictionary were fixed to 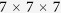 and 50,000, respectively, and LLC was used in local linear representation. Results show that a larger number of training subjects produce better results. We attribute this performance to the fact that a larger number of training subjects generate more representative dictionary. Thus, 20 training subjects were selected to be the training dataset in the subsequent experiments.

#### Parameter setting for the size of patch and dictionary

**Figure 4:**
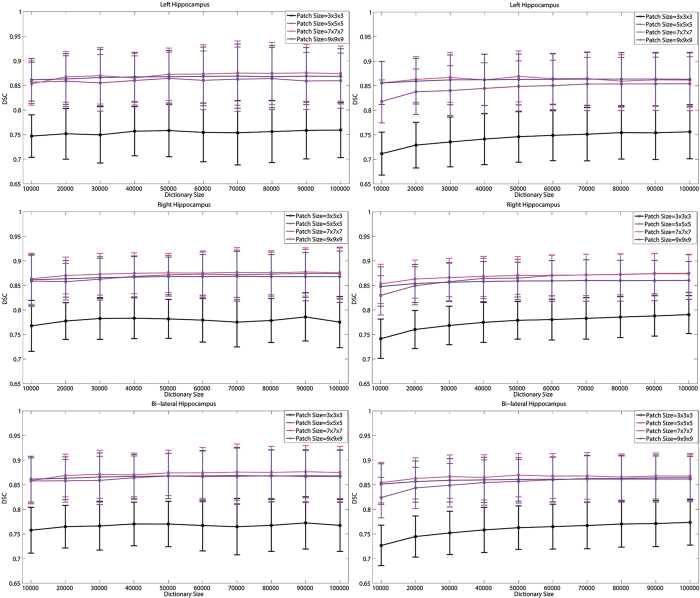
Mean DSC and standard deviation for the segmentation of left (top row), right (middle row), and bi-lateral (bottom row) hippocampus over different sizes of patch and dictionary using LLC (left column) and LAE (right column) in local linear representation.

Figure 4 shows the mean DSC for the segmentation of the left, right, and bi-lateral hippocampus over different sizes of patch and dictionary using LLC and LAE in local linear representation. Patch sizes (Unit: mm^3^) ranged from 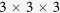 to 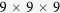 with an interval of 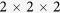. Dictionary sizes varied from 10,000 to 100,000 with an interval of 10,000. As shown in Fig. 4, DSC increases significantly from 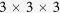 patch size to the 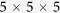, which results from the fact that similar small MR patches could correspond to distinct DF patches and Assumption II is violated. Too large size of patch could result in large distance between the patches in 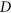, thus Assumption I is difficult to ensure and the DSC of bi-lateral hippocampus segmentation decreases with the patch size increasing from 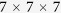 to 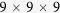. The result showed that a patch size of 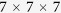 was a good choice for LLC and LAE. The accuracy of LLC was improved by increasing the size of dictionary. However, improvement plateaued when the size of dictionary exceeded 70,000. Large dictionary size indicated high memory and computation costs. The sizes of the dictionary and patch for LLC were set to 70,000 and 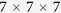, respectively. In LAE, with the dictionary size increasing, the accuracy of the left hippocampus declined and improvement of accuracy for the right hippocampus plateaued when the size of dictionary exceeded 50,000. Therefore, the optimal sizes of dictionary and patch for LAE are 50,000 and 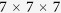, respectively.

#### Method selection in local linear representation

Given that LLC and LAE emphasize the locality in local linear representation, we performed the experiments using these methods to select an optimal method. Table 1 shows the mean DSC and standard deviation of left, right, and bi-lateral hippocampus segmentation using LLC and LAE. As shown in Table 1, LLC significantly improved the accuracy compared with LAE, in which the average DSC increased by 0.61%, 0.60% and 0.60% for left, right, and bi-lateral hippocampus, respectively. Therefore, LLC was selected for local linear representation in subsequent experiments.

#### Contribution of CWA

**Figure 5:**
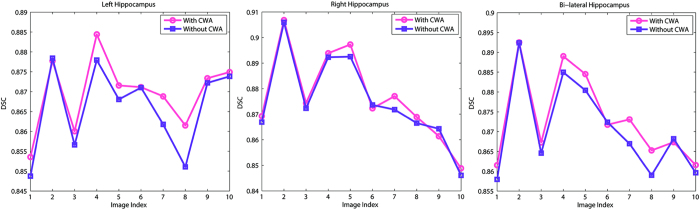
DSC of left, right, and bi-lateral hippocampus for 10 test subjects using methods with CWA and without CWA.

Two groups of experiments were performed to verify the effectiveness of CWA. In the first group (denoted as With CWA), we performed the experiment using the proposed method called LLM with CWA. In the other group (denoted as Without CWA), for a point 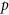 in the test image, we obtained a series of predicted DF values via Eq. (2) from the overlapped patch and the average of these predicted values was used as the final estimator for point 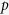. Figure 5 lists the DSC of 10 test subjects. Figure 5 shows that the method with CWA outperformed the method without CWA for most of subjects. Table 2 demonstrates the mean DSC and standard deviation of left, right, and bi-lateral hippocampus for 10 test subjects using methods with CWA and without CWA. Compared to method without CWA, the mean DSC of left, right, and bi-lateral hippocampus of method with CWA increased significantly (paired t-test *p* = 0.08, 0.049 and 0.009, respectively) by 0.37%, 0.18% and 0.28%, respectively.

### Comparison with Relevant Methods

To investigate the contribution of LLM, we compared the proposed method with several state-of-the-art label fusion algorithms and distance field fusion method, namely, majority voting^18^, SIMPLE^31^, STAPLE^27^, spatial STAPLE^20^, and DFF^29^. Label fusion algorithms (majority voting, SIMPLE, STAPLE, and spatial STAPLE) were performed using MASI Label Fusion toolbox (http://www.nitrc.org/projects/masi-fusion) with default parameters. Table 3 shows the mean ± standard deviation of DSC for the left, right, and bi-lateral hippocampus using different segmentation methods. The mean ± standard deviation of DSC obtained by the proposed method was 0.8697 ± 0.0091 for the left hippocampus, 0.8770 ± 0.0176 for the right hippocampus, and 0.8734 ± 0.0113 for the bi-lateral hippocampus. Results showed that the proposed method generated higher accuracy than those of the four relevant label fusion algorithms. Compared to DFF, the segmentation accuracy of LLM decreased by 0.31%, 0.55% and 0.43% for left, right, and bi-lateral hippocampus, respectively. However, DFF is a multi-atlas based method that requires deformable registration. Given the trade-off between memory and computation costs and accuracy, the performance of the proposed method LLM was comparable to that of DFF.

**Figure 6:**
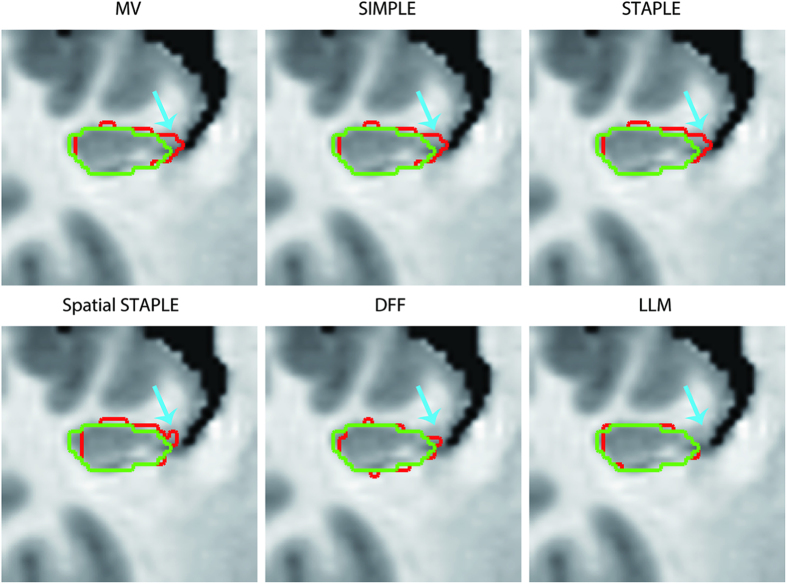
Coronal view of segmentation results for the right hippocampus of a test subject using our method and five other methods, namely, majority voting (MV), SIMPLE, STAPLE, spatial STAPLE and DFF, along with the manual segmentation. Red and green contours denote the automatic and manual segmentation results, respectively.

Figure 6 shows the coronal view of segmentation results for the right hippocampus of a test subject using our method and five other methods. The hippocampus segmented by our method was more similar to the ground-truth than in any other methods (i.e., majority voting, SIMPLE, STAPLE, spatial STAPLE and DFF). Particularly, in the arrow region, our method could accurately delineate the boundary of the hippocampus, whereas the five other methods led to large errors.

## Discussion

In this study, we propose a novel LLM-based method to segment hippocampus for MR image. The main contributions of this study are as follows. First, we utilize label information and distance information from the boundary of the hippocampus to improve the segmentation accuracy of LLM. Second, we assume that MR image patches and DF patches are located on nonlinear manifolds, and the mapping between these manifolds is locally linear and differentiable. The fusion weights of DF patches can be deduced from the weights of MR image patches. Third, according to Assumption II, a novel k-means based method is proposed to build a compact dictionary that ensures the accuracy with limited computation and memory costs. Fourth, CWA is proposed to facilitate fusion of the overlapped prediction of DF. Segmentation accuracy is significantly improved by the residual of local linear representation.

In our previous study^43^, the dictionary was constructed by considering the locality of image space (i.e. constructing the dictionary using the training samples around the test point in image space) and LAE was superior to LLC. However, in the current study, we neglected the locality of image space and LLC outperformed LAE. We cannot provide a complete explanation for this phenomenon, but we assume that it is caused by the introduction of locality of image space in the training dataset.

In the current study, we conduct the experiments for T1-weighted MR brain image. In clinical setting, multi-modality image analysis is imperative for accurate diagnosis. Conducting a comparison with different modalities may provide a better understanding of the effectiveness of the proposed method in clinical setting. In our future study, we will collect more datasets of multiple modalities to expand the proposed method to multi-modality image analysis.

Computation time can be improved. We use a single thread to perform our experiments. In our method, we predict DF patches point-by-point. The procedure for predicting DF patches at different points is independent, which provides the possibility for parallel computation. In future studies, parallel computation by multi-thread and GPU computation will be used to improve the speed of segmentation. Information on patch similarity and the feature distinctness should be incorporated to enhance segmentation performance^44^,^45^. Moreover, as mentioned in Section “Introduction”, changes in the size and shape of hippocampus are closely related to Alzheimer’s and other mention disorders, we will calculate the volume of hippocampus using the proposed method and study the relation between the size of hippocampus and Alzheimer’s in our future work.

In conclusion, this study presents a novel method for hippocampus segmentation based on LLM. The proposed method is compared with majority voting, SIMPLE, STAPLE, spatial STAPLE, and DFF in public datasets. The accuracy of the proposed method is higher than the first four aforementioned label fusion methods and comparable with DFF.

## Additional Information

**How to cite this article:** Pang, S. *et al*. Hippocampus Segmentation Based on Local Linear Mapping. *Sci. Rep.* **7**, 45501; doi: 10.1038/srep45501 (2017).

**Publisher's note:** Springer Nature remains neutral with regard to jurisdictional claims in published maps and institutional affiliations.

## Figures and Tables

**Table 1:** Mean DSC and standard deviation of left, right, and bi-lateral hippocampus segmentation for LLC and LAE.

Methods in local linear representation	Left	Right	Bi-lateral
**LLC**	**0.8751 ± 0.0246**	**0.8763 ± 0.0179**	**0.8757 ± 0.0207**
**LAE**	0.8690 ± 0.0276	0.8703 ± 0.0191	0.8697 ± 0.0230

**Table 2:** Mean DSC and standard deviation of left, right, and bi-lateral hippocampus for 10 test subjects using methods with CWA and without CWA.

Methods	Left hippocampus	Right hippocampus	Bi-lateral hippocampus
**With CWA**	**0.8697 ± 0.0091**	**0.8770 ± 0.0176**	**0.8734 ± 0.0113**
**Without CWA**	0.8660 ± 0.0108	0.8752 ± 0.0172	0.8706 ± 0.0118

**Table 3:** Mean ± standard deviations of the evaluation metrics for the left hippocampus using the proposed method and five relevant methods.

Methods	Left Hippocampus	Right Hippocampus	Bi-lateral Hippocampus
**Majority voting**	0.8520 ± 0.0216	0.8582 ± 0.0254	0.8552 ± 0.0226
**SIMPLE**	0.8515 ± 0.0224	0.8589 ± 0.0248	0.8553 ± 0.0227
**STAPLE**	0.8522 ± 0.0252	0.8552 ± 0.0286	0.8537 ± 0.0261
**Spatial STAPLE**	0.8529 ± 0.0241	0.8579 ± 0.0268	0.8554 ± 0.0247
**DFF**	**0.8728 ± 0.0115**	**0.8825 ± 0.0140**	**0.8777 ± 0.0119**
**LLM**	0.8697 ± 0.0091	0.8770 ± 0.0176	0.8734 ± 0.0113
